# CRISPRi-mediated transcriptional silencing in iPSCs for the study of human brain development

**DOI:** 10.1016/j.xpro.2022.101285

**Published:** 2022-04-13

**Authors:** Pia Annette Johansson, Anita Adami, Johan Jakobsson

**Affiliations:** 1 Laboratory of Molecular Neurogenetics, Department of Experimental Medical Science, Wallenberg Neuroscience Center and Lund Stem Cell Center, Lund University, 221 84 Lund, Sweden; 2 Cell and Gene Therapy Core, Lund Stem Cell Center, Lund University, 221 84 Lund, Sweden

**Keywords:** Developmental biology, CRISPR, Neuroscience, Stem Cells, Cell Differentiation

## Abstract

This protocol describes the design and use of CRISPRi-mediated transcriptional silencing in human iPSCs, for loss-of-function studies in brain development research. The protocol avoids single cell selection, thereby eliminating side effects of clonal expansion and sites of viral integration. We also describe a neural progenitor differentiation protocol and discuss the challenges of target-specific lentiviral silencing, efficient silencing levels, and off-target effects.

For complete details on the use and execution of this protocol, please refer to Johansson et al. (2022).

## Highlights

• Guidelines for selection of CRISPRi-guides via CRISPick and USCS Genome Browser
• Protocol for viral transduction of human iPSCs
• Protocol for differentiation of iPSC into forebrain neural progenitor cells

## Graphical abstract

**Figure:**
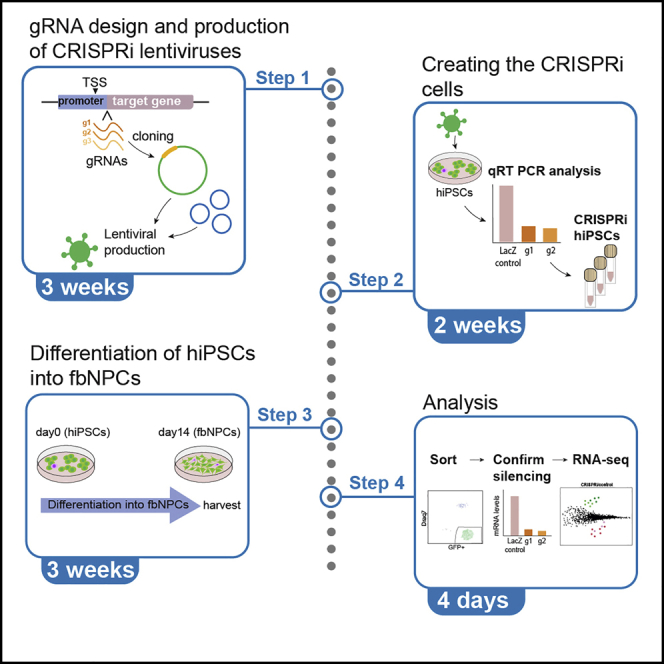

## Before you begin

The protocol below describes the specific steps for using CRISPRi in human induced pluripotent stem cells (hiPSCs). This protocol has also been used in fibroblasts converted to induced neurons (Pircs et al., 2022) and is likely suitable for other cell lines. We have used an all-in-one CRISPRi backbone from Addgene (Thakore et al., 2015; Addgene_71237). Molecular cloning, lentiviral production, qRT-PCR and hiPSCs culture techniques need to be established in the lab for successful application of this protocol (these techniques are not covered here). Verified qRT-PCR primers for the gene of interest and one or two housekeeping genes are also essential.

### Design, cloning, and production of CRISPRi lentiviruses

**Timing: 3 weeks**

Before establishing the CRISPRi system, it is necessary to design the guide RNAs for the chosen target gene(s), clone them into the correct plasmid backbone, and finally produce the lentiviral vectors that will be used for the transcriptional silencing in iPSCs.

**Figure 1:**
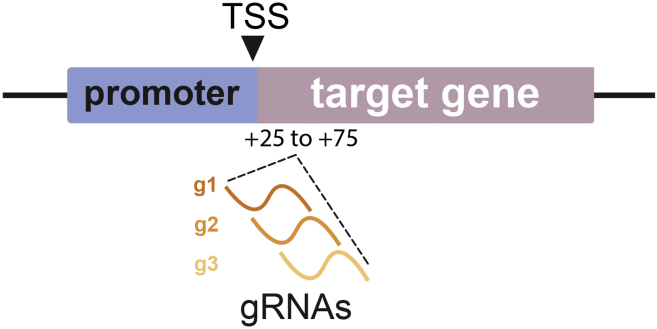
Schematic of the region the guide RNAs target CRISPick automatically considers the region 25–75 bp downstream of the TSS of the gene of interest and designs the guide RNAs against a sequence within that region.

1. Order and make maxiprep of CRISPRi cloning backbone from Addgene (Addgene_71237).
2. Design 3 guide RNA (gRNA) sequences for the gene of interest according to the CRISPick portal (https://portals.broadinstitute.org/gppx/crispick/public). The software automatically selects guide RNAs that target a region +25–+75 bp downstream the transcription start site (TSS) of the target gene (Figure 1 and troubleshooting 1). See also “CRISPick: How It Works”.
  a. Choose a reference genome (Human GRCh38), mechanism (CRISPRi), and Enzyme (SpyoCas9).
  b. Type in the gene of interest.
  c. Tick the "report unpicked sequences" box.
  d. Wait for the software to generate the reports.
  e. Download the “Picking Results” .txt file.
  f. Open with Excel, scroll to Column AR and find the software’s preferred picks.
  g. Choose 3 guides for each gene of interest.
  h. The gRNA sequence is in column S.

**CRITICAL:** In order to minimize the effects of guide-specific off-targets, at least two working guide RNAs are needed (see more below).

***Note:*** A minimum of 3 guides are always designed for each gene. The success rate of the gRNA varies between different genes. While many genes are efficiently silenced with most tested guides, some genes are more challenging to target. In these instances, many guides may have to be tested in order to achieve sufficient silencing.

***Note:*** Consider what level of silencing would be sufficient for the experiment, it is not always critical nor desired to achieve 99% knock-down efficiency.

***Optional:*** Investigate the potential off-targets of the designed gRNAs. See troubleshooting 5.

**Figure 2:**
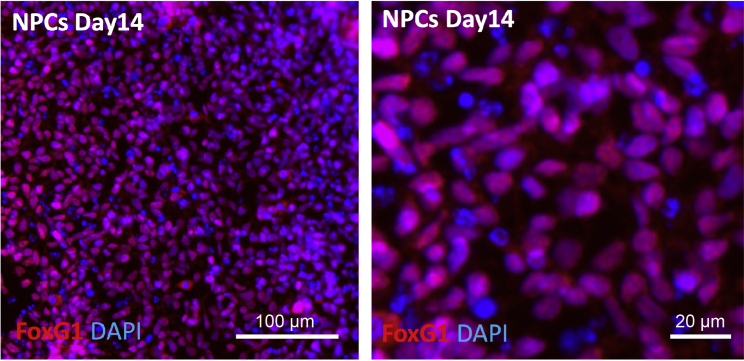
Representation of the investigation of guides, isoforms and alternative TSSs

***Optional:*** Investigate the position of the guides and the predicted TSS in the USCS genome Browser https://genome.ucsc.edu/cgi-bin/hgGateway. This is shown below using ZNF248 (simplified schematic, Figure 2) as an example.

Select the human GRCh38 assembly (Figure 2, red circle) and type in the name of your gene of interest for a first view (Figure 2, blue circle). Then, type in the predicted TSS from column K in the CRISPick design sheet using this format: chr10:37857610 (green circle). This will show one nucleotide, highlight it and zoom out to see its location in the gene. In the ZNF248 example, the predicted TSS (dark green line) falls at the start of two main isoforms.

Once you highlighted the nucleotide and zoomed out, type in the first gRNA sequence. The browser will show the number of places it finds this sequence (which should only be one). Click on “browser”(Figure 2, pink circle) and get to the sequence that can now be highlighted. Do the same with the other gRNA target sequences.

In the ZNF248 example, the selected guides are found downstream of the TSS (ZNF248 runs in anti-sense direction) and can be seen to have some sequence overlap (this is often unavoidable as the target window is quite small) (Figure 2). However, there is one isoform (Figure 2, red arrow) which might have an alternative TSS. This needs to be kept in mind if one does not know which isotype is expressed. However, in the given example, the RNA-sequencing tracks from the hiPSC-derived forebrain neural progenitor cells (fbNPCs, our cells of interest, Johansson et al., 2022) show no evidence of transcription of the isoform-specific exon (red rectangle), suggesting that the designed CRISPRi guides should be sufficient for silencing all the expressed isoforms. If there is evidence of a different TSS for some isoforms and it is not possible to tell which isoforms are expressed in the cells of interest, then first use the TSS defined by the software. If the transcriptional silencing is not sufficient, then consider manually designing guides for the alternative TSS and combine the two guides (see troubleshooting 4).

3. Order the oligos with specific overhangs for BsmBI cloning. Insert the 20 bp target gRNA sequence between the overhangs.

Forward oligo: 5′ CACCG…….20 bp target………-3′

Reverse oligo: 5′ AAAC……20 bp………C 3′

**CRITICAL:** Use a non-targeting guide (e.g., against the bacterial LacZ) as a control for all experiments. Order oligos for this sequence as well (See key resources table).

4. Insert the guides into the CRISPRi lentiviral backbone (Figure 3) containing both the guide RNA under the U6 promoter and dead-Cas9-KRAB and GFP under the Ubiquitin C promoter (pLV hU6-sgRNA hUbCdCas9-KRAB-T2a-GFP, RRID:Addgene_71237; Thakore et al., 2015). See here for PDF cloning protocol, part A (automatic download).

**Figure 3:**
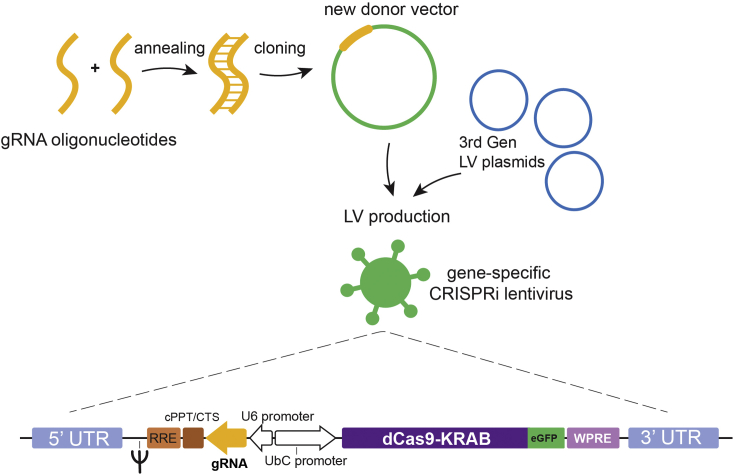
Workflow for lentiviral production After designing of the desired guide RNAs, the resulting oligonucleotides (yellow) are annealed and cloned into the backbone of the CRISPRi plasmid (green), which is then used, along with standard 3^rd^ generation lentiviral plasmids (blue), to produce the gene specific CRISPRi lentiviral vector.
5. Lentiviral vectors were produced according to (Zufferey et al., 1997) and were in titers of 10^8^ - 10^9^ TU/mL as determined by qPCR of genomic DNA after serial dilution transductions. We recommend using an MOI between 5 and 15.

**CRITICAL:** To obtain high transduction and high silencing efficiency, it is crucial to obtain a high titer.

## Key resources table

**Table:** 

REAGENT or RESOURCE	SOURCE	IDENTIFIER
**Antibodies**		

Anti-rabbit FOXG1, polyclonal 1:75 dilution	Abcam	Cat# ab18259 (RRID: AB_732415)
Anti-rabbit PAX6, polyclonal, 1:500 dilution	BioLegend	Cat# 901301 (RRID: AB_2565003)
Anti-rabbit CY3, secondary antibody, 1:200 dilution	The Jackson Laboratory	n/a

**Chemicals, peptides, and recombinant proteins**		

Ascorbic Acid	Sigma-Aldrich	Cat# A5960
B27 (-Vit A) supplement	Gibco (Thermo Fisher Scientific)	Cat# 12587010
DMEM/F-12	Gibco (Thermo Fisher Scientific)	Cat# 21331020
Draq7	BD Biosciences	Cat# 564904
Knockout replacement serum (KSR)	Gibco (Thermo Fisher Scientific)	Cat# 10828010
L-Glutamine	Gibco (Thermo Fisher Scientific)	Cat# 25030032
Laminin-111	BioLamina	LN111
Laminin-521	BioLamina	LN521
N2 supplement	Gibco (Thermo Fisher Scientific)	Cat# 17502048
Neurobasal	Gibco (Thermo Fisher Scientific)	Cat# 21103049
Noggin	Miltenyi Biotec	Cat# 130-103-456
Penicillin-Streptomycin	Gibco (Thermo Fisher Scientific)	Cat# 15140122
rhBDNF	R&D	Cat# 248-BD
Rock-inhibitor, Y27632	Miltenyi Biotec	Cat# 130106538
SB431542	Axon	Cat# 1661
StemMACS iPS-Brew XF, human	Miltenyi Biotec	Cat# 130104368
StemPro Accutase	Gibco (Thermo Fisher Scientific)	n/a

**Experimental models: Cell lines**		

Human iPSC line (HS1), female, >1D	RIKEN	RBRC-HPS0328 606A1 (RRID:CVCL_DQ11)

**Oligonucleotides**		

sgRNA: ZNF138-g1 GG GTCTTTGTCTCGCTGCAG	Johansson et al., 2021	n/a
sgRNA: ZNF138-g2 CTGCG TCCTCTTACTCCTAG	Johansson et al., 2021	n/a
sgRNA: ZNF248-g2 AATTA GACACAATTATCCAC	Johansson et al., 2021	n/a
sgRNA: ZNF248-g3 ACA ATTATCCACGGGAGACT	Johansson et al., 2021	n/a
sgRNA: ZNF558-g2 GCCA AAAGCGCCGACTCGCG	Johansson et al., 2021	n/a
sgRNA: ZNF558-g3 AGTCG GCGCTTTTGGCCCCG	Johansson et al., 2021	n/a
sgRNA: LacZ TGCGAATA CGCCCACGCGAT	Johansson et al., 2021	n/a
ZNF138 fw TGGCGCTGTGA TCTGGTTATT rev CAGTGG TCCCATTTCTAGGCTT	this paper	n/a
ZNF248 fw GTGCCACCCAG AAAGGAAATG rev AGGGT CTGTGCCCAAATTGAA	this paper	n/a
ZNF558 fw CGGGCTCGAT GAGAAAAGCA rev ATTCT CACACACTGCTGGGG	Johansson et al., 2021	n/a

**Recombinant DNA**		

Plasmid: pLV hU6-sgRNA hUbC-dCas9-KRAB-T2a-GFP	Thakore et al. (2015)	RRID: Addgene_71237

**Software and algorithms**		

n/a	n/a	n/a
n/a	n/a	n/a

## Materials and equipment

None of the following media has been filtered when prepared.

**Table:** iPSBrew∗

Component	Storage	Dilution
iPS-Brew XF, Basal Medium	−20°C	1:1
iPS-Brew XF, 50× Supplement	−20°C	1:50
Penicillin/streptomycin (stock: 10,000 U/mL)	−20°C	0.5%

∗This is the only iPSC media that we used, although we can only speculate, we do not expect the use of other media to influence CRISPRi efficiency.

**Table:** N2 media

Component	Storage	Dilution	Amount for 50 mL
DMEM/F12	4°C	49%	24.5 mL
Neurobasal	4°C	49%	24.5 mL
N2 supplement (100×)	−20°C	1:100	500 μL
L-Glutamine (stock: 200 mM)	4°C	1:100	500 μL
Penicillin/streptomycin (stock: 10,000 U/mL)	4°C	1:500	100 μL
**Total**	**n/a**	**n/a**	**50 mL**

Make fresh every 2 weeks. Add small molecules on the day of feeding.

**Table:** B27 media

Component	Storage	Dilution	Amount for 50 mL
Neurobasal	4°C	97%	48.5 mL
B27 (- vitamin A) (50×)	−20°C	1:50	1 mL
L-Glutamine	4°C	1:100	500 μL
Penicillin/streptomycin (stock: 10,000 U/mL)	4°C	1:500	100 μL
**Total**	**n/a**	**n/a**	**50 mL**

Make fresh every 2 weeks. Add small molecules on the day of feeding.

**Table:** FACS media (fbNPCs)

Reagent	Dilution	Amount
B27 media w/o small molecules∗∗		0.5 mL/sample
Draq7	1:1,000	0.5 μl/sample
Rock Inhibitor (stock: 10 mM)	1:1,000	0.5 μl/sample
**Total**	**n/a**	**n/a**

**NB** Make fresh on the day.

∗∗If you choose to sort iPSC then exchange the B27 media for iPSC Brew.

## Step-by-step method details

### Significance of not using clonal expansion

When using lentiviral delivery, the lentiviral units will integrate into the genome at different sites, some of which can lead to a phenotypic alteration in the cell. Additionally, clonal expansion of isogenic clones can lead to alterations in the transcriptome (Carcamo-Orive et al., 2017). If one uses cells of mixed clonal origins, the effect of viral integration and clonal expansion will be reduced and the signal to noise ratio of transgene expression will be increased.

### Establishing the CRISPRi cells

**Timing: 2 weeks**

In this step, human iPSCs are transduced with the previously designed CRISPRi lentiviral vector. This results in the creation of cells with a constitutive downregulation of the target gene.

**Figure 4:**
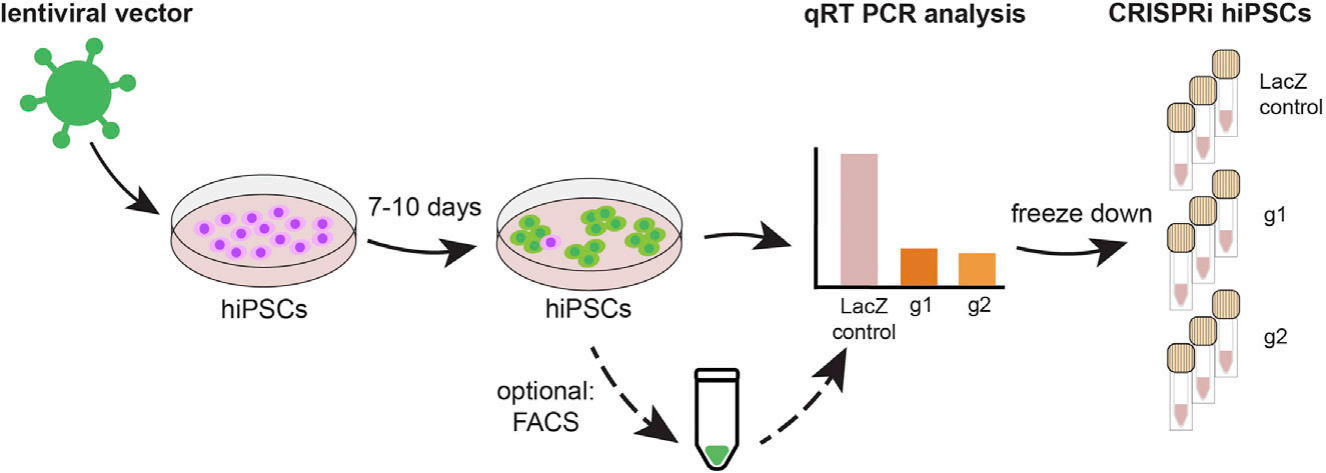
Schematic of the CRISPRi hiPSCs transduction After lentiviral transduction, the hiPSCs are grown for 7–10 days. Then, one can optionally FACS the GFP+ cells or directly proceed to check the efficiency of the transcriptional silencing. Once the successful CRISPRi has been confirmed, the cells are frozen down and ready to be used for the desired experiment.

1. Creation of CRISPRi cells (Figure 4).
  a. Culture hiPSCs on Laminin521 (Lam521)-coated wells and feed everyday with IPSBrew. Use 900 μL PBS +/+ and 25 μL of Lam521 (0.5 μg/cm^2^) and incubate in the fridge for 12–16 h (or at 37°C for 2 h). Split every 3–4 days with Accutase (incubation for 10 min at 37°C), seeding 100,000–200,000 cells per well in a 12-wells plate with 900 μL of iPSBrew. (See Grassi et al., 2019; Johansson et al., 2022).
  b. On the day of transduction, split the cells with Accutase (incubation for 10 min at 37°C). Count the cells.
  c. Seed 200,000 cells per well in a 12-wells plate. Use one well per condition. Use double amount of media (1.8 mL) with 10 μM of Rock inhibitor (RI).
  d. Plate some untransduced cells as negative control.
  e. Directly after seeding the cells, dilute each virus 1:10 in iPSBrew and add the equivalent of MOI10 to each well (the first time, use MOI5 and 15 as well to select the best MOI for the virus and cell line combination). Add one virus per well.
  f. Change the media every day and add 10 μM Rock Inhibitor until the day after the first split (∼70% confluency). The first split ideally takes place on day 3 post-transduction.
  g. On the 3^rd^ day post-transduction, split cells with Accutase (incubation for 10 min at 37°C). They should now behave quite normally.
  h. On day 7–10 split again with Accutase (incubation for 10 min at 37°C), collect cells, and take samples for RNA and cryopreservation. If, instead, very little cells have survived the transduction, then see troubleshooting 3.
    i. **RNA**: Collect 200,000 cells, spin down, remove media and snap-freeze.
    ii. **Cryopreservation**: Freeze 3–4 vials of cells for further experiments.
  i. Seed for start of differentiation (optional).
  j. Extract RNA, synthesize cDNA and check CRISPRi silencing efficiency using qRT-PCR for all three guides. We use the delta Ct method with the LacZ control virus as the nominator (set as 1 to normalize the calculation) for analysis.
  k. Select the two guides that produces the most efficient silencing for further studies. Ideally, the silencing efficiency is ≥ 90%.
    ***Optional:*** FACS GFP+ cells prior to investigating silencing efficiency and freezing. This is preferable if the transduction efficiency is low. If feasible, FACS at the endpoint of the experiment is still recommended in case of low levels of silencing or selection during differentiation. If low downregulation levels are reached with all of the designed guides, see also troubleshooting 4.
    ***Note:*** Selection via FACS is needed at either the start or end of experiment. Selection at the start gives a better indication of actual silencing efficiency and it is often better for long-term experiments to start with a purer population. Selection at the end is not always possible due to complex differentiation protocols (for instance, with cerebral organoids the association into single cells needed for selection via FACS destroys some cell populations). In case of fbNPC (described here), we sorted only at the end of the experiment as these cells are well suited for single cell dissociation. For cerebral organoid differentiated described in Johansson et al., (2022), we sorted the cells prior to start of differentiation and then used single-nuclei dissociation for analysis. If selection via FACS is not possible, puromycin selection can be an alternative method to establish the CRISPRi cells (see troubleshooting 2).
    ***Note:*** We recommend freezing serval vials of cells early in procedure to be able to use similar passage numbers for repeat or follow-up experiments as well as to avoid a decrease in efficiency (due to silencing and/or uneven growth patterns of transduced and un-transduced cells) with increased passage number and repeated freeze-thaw cycles. In our hands we have not seen much effect of these processes, but this can also depend on the gene of interest.
    ***Note:*** We have not tested other culture media or coating matrices but anticipate that other media or matrices will not significantly affect the procedure.

### Differentiate hiPSCs into fbNPCs

**Timing: 3 weeks**

**Figure 5:**
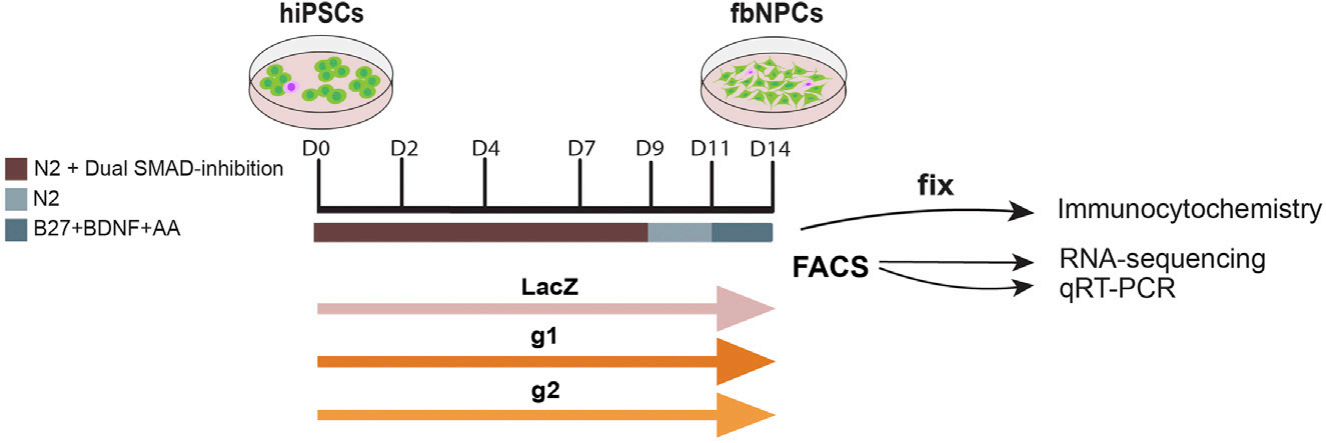
Schematic of the differentiation protocol used in this project The differentiation is a two-week long protocol based on dual-SMAD inhibition that allows differentiation of hiPSCs into fbNPCs.

Here, the steps to differentiate the hiPSCs into forebrain NPCs (fbNPCs) are described (Figure 5, Grassi et al., 2019 and Johansson et al., 2022). The CRISPRi cells have also been differentiated into cerebral organoids (see Johansson et al., 2022) with efficient silencing shown to persist after four months, see also expected outcomes.

**Figure 6:**
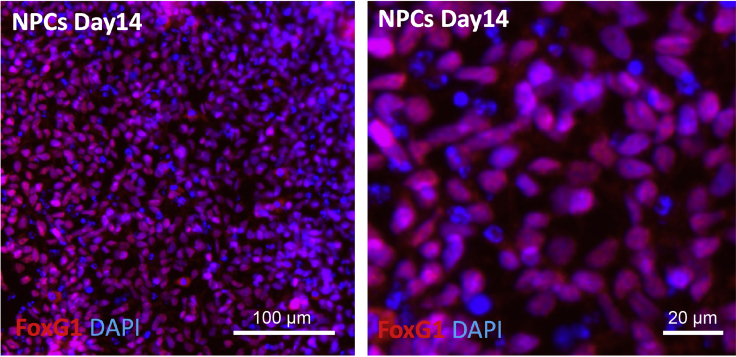
Immunocytochemistry of FOXG1 in fbNPCs On day 14, the fbNPCs were fixed and then stained for FOXG1 (red) and DAPI (blue).
  c. Collect and sort the cells.
    i. Prepare FACS media and collection media (B27 media + Rock Inhibitor (10 μM).
    ii. Collect the cells using Accutase (10 min incubation at 37°C) to ensure single cell suspension.
    iii. Resuspend the cells in FACS media and filter prior to FACS.
    iv. Prepare collection tubes with collection media (B27 media + Rock Inhibitor (10 μM).
    v. Sort GFP+ cells into the collection tube.
    vi. Keep on ice.
    vii. Spin down, remove supernatant and snap-freeze pellet and store at −80°C.

2. Thaw the two CRISPRi cell batches with the most efficient transcriptional silencing (e.g., guide1 and guide2), the LacZ CRISPRi control (experimental control), and include one untransduced control (FACS control).
3. Split the cells once before the start of differentiation.
4. Coat one well in a 6-well plate (2.5 mL PBS + 150 μL Lam111) per condition. Leave 12–16 h at 4°C.
5. Day 0.
  a. Collect the cells from four different conditions and count the number of cells.
  b. Seed 75,000 cells onto each Lam111-coated well in N2 media + Noggin (100 ng/mL) + SB431542 (10 μM) + Rock Inhibitor (10 μM) from each condition.
6. Day 2. Replace media (N2 media + Noggin (100 ng/mL) + SB431542 (10 μM)), 2.25 mL/well. As the cells grow more dense, the volume of media will increase throughout the differentiation.
7. Day 4. Replace media (N2 media + Noggin (100 ng/mL) + SB431542 (10 μM)), 2.5 mL/well.
8. Day 7. Replace media (N2 media + Noggin (100 ng/mL) + SB431542 (10 μM)), 3 mL/well.
9. Day 9. Replace media, N2 media only, 3.5 mL/well.
10. Day 10. Coat 12-well plate with Lam111 (900 μL PBS + 60 μL Lam111, 1 well per condition), and 96-well plate with 100 μL PBS + 6 μL Lam111 (2–3 wells per condition).
11. Day 11. Split cells with Accutase (10 min incubation at 37°C°) and reseed (3 million cells/well) in B27 media + AA (0.2 mM) + BDNF (20 ng/mL) + RI (10 μM). Seed also 250,000 cells per condition per well in a 96-well plate for immunocytochemistry.
12. Day 14**.** On day 14 the cells are fbNPCs as defined by FOXG1 and PAX6 staining and their transcriptional profile (Grassi et al., 2019, Johansson et al., 2022).
  a. Fix cells in the 96-wells plate for 15 min at RT using ice-cold 4% paraformaldehyde.
  b. Perform immunocytochemistry of FoxG1 (1:75, RRID:AB_732415) for confirmation of forebrain identity (Figure 6). These cultures are homogenous at day 14 and almost all cells should appear positive after immunohistochemistry. Very similar results have been found with another human iPSC lines, one human ES line and two chimpanzee iPSC lines (Grassi et al., 2019; Johansson et al., 2022). Additionally, when we performed single cell RNA-seq of these cultures, 95% of the analyzed cells were found in one main Pax6 and FoxG1 positive cluster (Johansson et al., 2022).

**CRITICAL:** Bring untransduced cells to the FACS to set the gate for GFP and Draq7 (cell death marker).

***Note:*** As the cell density increases the protocol calls for increased volume of media.

***Note:*** One sample is needed for RNA isolation (RNeasy Mini kit (50) (QIAGEN, cat. # 74104)) to re-assess the transcriptional silencing efficiency at the end of the experiment. Collect additional samples depending on the scientific question and assays. We typically collect 3 × 200,000 cells per condition.

***Optional:*** If the gene of interest encodes for a protein the silencing can also be validated using WB.

Silencing can also be validated using RNA-sequencing, via histone methylation marks-mediated CUT&RUN or ChIP, where reduction of H3K4me3 and/or enrichment of H3K9me3 markers confirm the silencing.

### Combine guides for analysis for reduction of off-target effects

**Timing: 4 days**

**Figure 7:**
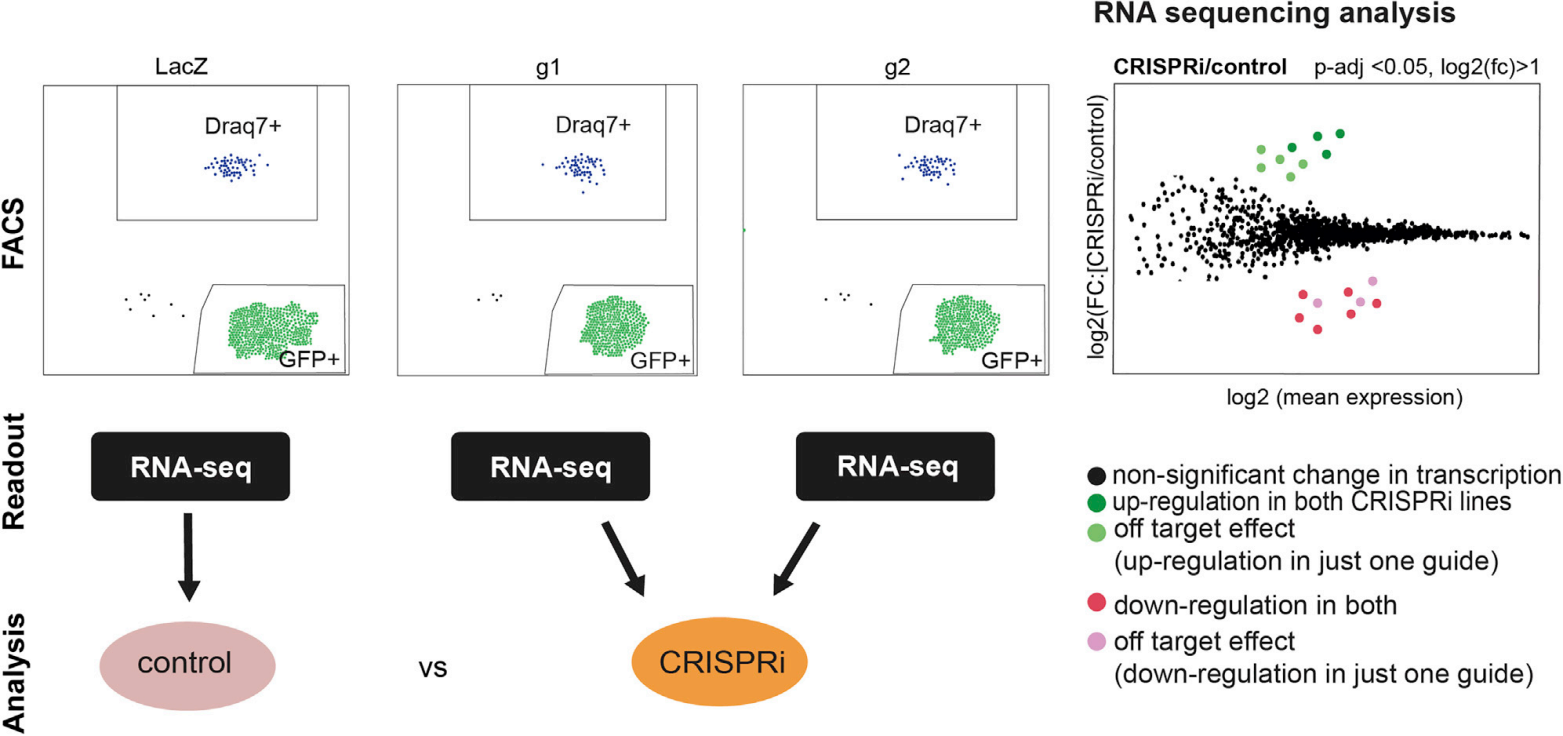
Schematics of fbNPCs FACS and RNA-seq analyses Once the GFP+ fbNPCs are sorted via FACS, they are prepared for RNA-sequencing. The sequencing results are then analyzed to compare transcriptional differences between the control cells and the CRISPRi cells.

This part of the protocol involves combining or verifying the effect of the CRISPRi using two different guides. This varies depending on the read-out. RNA-sequencing is used here as readout, and the combination of guides will be described below (Figure 7).

Most guides will have off-targets. However, two guides are very unlikely to have the same off-targets. Thus, combining the readouts of two guides will minimize the off-target effect of each guide. Here, we combine the two CRISPRi conditions into one, and analysis is performed with control vs CRISPRi cells (in our case n=4 and 8, respectively).

## Expected outcomes

**Figure 8:**
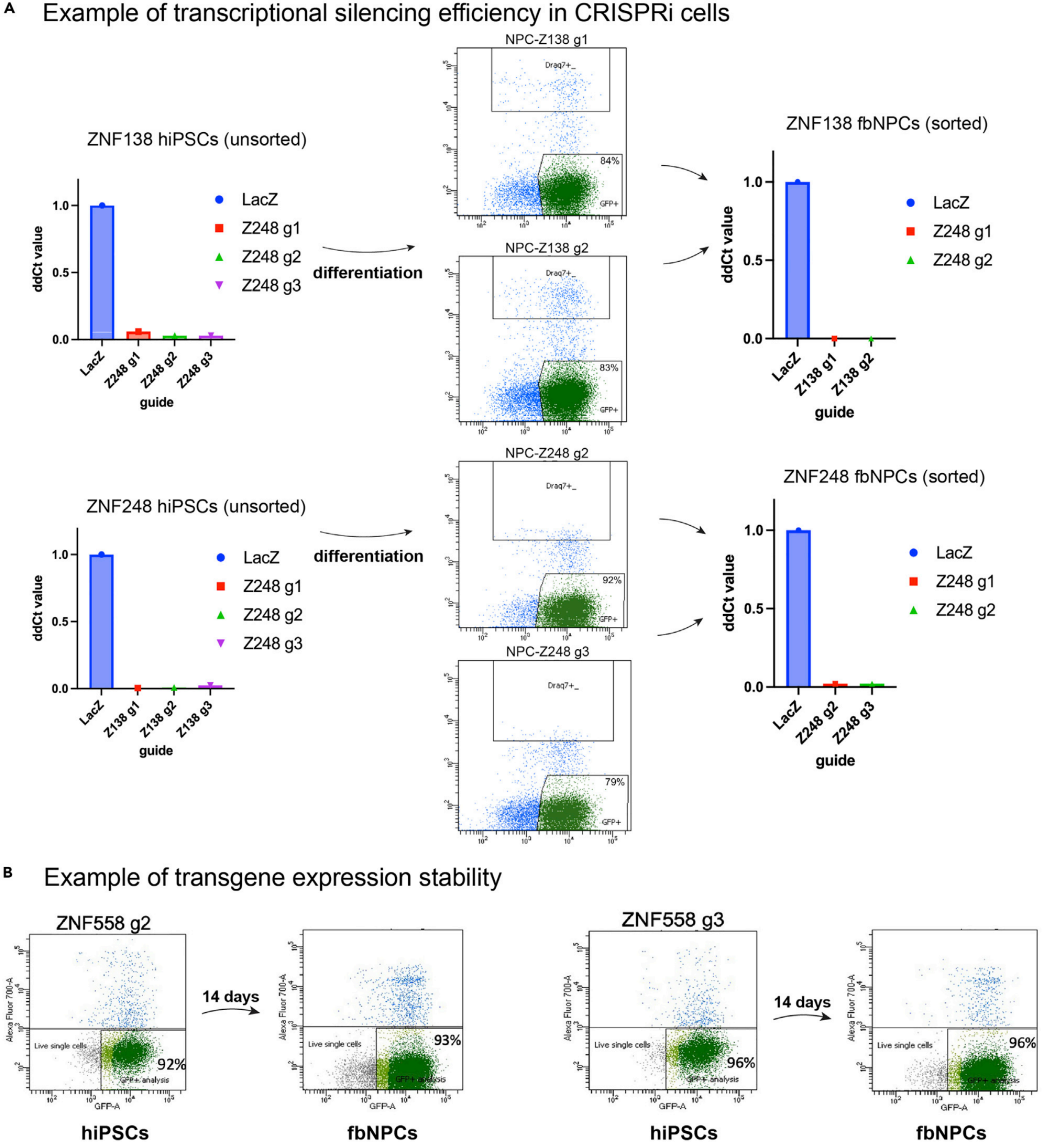
Examples of tranduction, silencing efficiency and transgene stability (A and B) Example of (A) transcriptional silencing efficiency and (B) transgene expression stability in CRISPRi cells.

Using this protocol, we generally get very high transduction efficiency (60–95%) and very high silencing efficiency (Figure 8A). Importantly for this protocol and the use of CRISPRi for long-term transcriptional silencing, the construct we have chosen is stably expressed, and after 2 weeks of differentiation we detected no reduction in the percentage of positive cells (Figure 8B). In cerebral organoids, we have stable transcriptional silencing even in 4-month-old organoids (Johansson et al., 2022).

***Note:*** A change in the percentage of positive cells may occur with increased time and/or differentiation due to phenotypic alterations (e.g., survival or proliferation) rather than transgene silencing.

## Troubleshooting

### Problem 1

*There is another gene close to my gene of interest running in the opposite direction* (See design, cloning and production of CRISPRi lentiviruses).

### Potential solution

This might not be a problem, CRISPRi works best when the Cas9 is guided to the DNA *downstream* of the transcription start site. However, there can still be interference. The solution is then to design a few more guides targeting around +75–+90 bp downstream of the transcription start site.

The CRISPRick software first chooses guides within the +25-+75 bp downstream of the transcription start site. However, one can manually pick guides outside of this range. Choose a guide that has a high pick order and comments are only “outside of range”. Design qRT-PCR guides for the nearby gene as well and test the effect at the same time as the gene of interest.

### Problem 2

*We cannot use GFP selection* (See establishing the CRISPRi cells).

### Potential solutions

• Use the sister construct Addgene Plasmid #71236), that has selection via puromycin.

We have not used this plasmid, but we anticipate it is equally efficient. We have however used puromycin in these cultures for selection of other vectors with a final concentration of 0.5 μg/μL. However, we have found the NPCs to be sensitive to puromycin (i.e., even with a puromycin resistance gene they thrive less) and we therefore recommend the user to do a robust selection during the iPSC stage and carefully optimize the puromycin selection during the differentiation procedure.

• Modify the plasmid to express RFP instead of GFP (also available from the lead contact).

### Problem 3

*There are not enough green cells after transduction* (See establishing the CRISPRi cells)*.*

### Potential solutions

**Figure 9:**
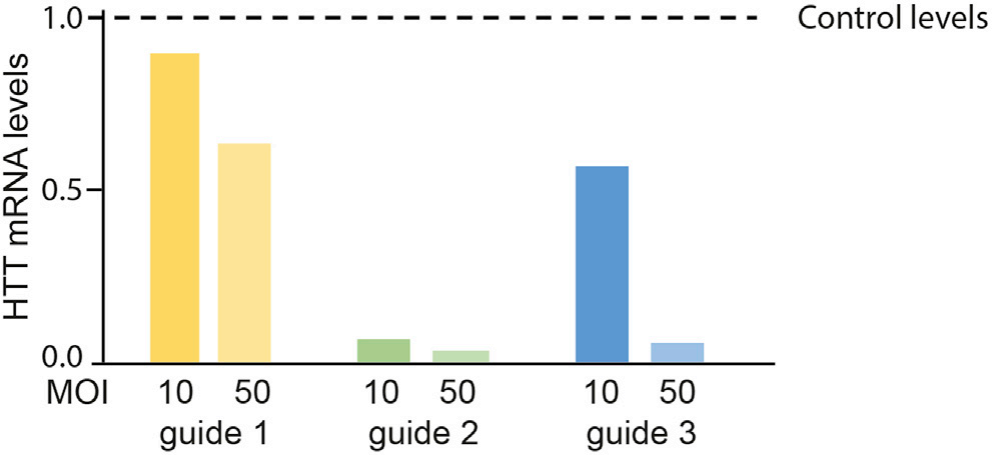
Effect of guide and MOI on silencing efficiency
• If the titre is low, try to modify production to achieve a higher titre.
• Expand the cells more before the sort and take cells from 2 or 3 wells to the FACS.
• Ensure the cells are not over-confluent when collecting for transduction.
• Use cells that have been split only once or twice after thaw.

• Use a range of MOIs to test for survival, transduction, and transcriptional silencing efficiency (Figure 9).

### Problem 4

*Not sufficient transcriptional silencing with either of the designed guides* (See Establishing the CRISPRi cells)*.*

We have found that the efficiency of CRISPRi is not only guide and position dependent, but also locus dependent. For example, in the case of ZNF558, CUT&RUN showed that the H3K4Me3 activation mark over the ZNF558 promoter was absent after ZNF558 CRISPRi (Johansson et al., 2022), indicating complete silencing. In other experiments, using the same methodology but targeting different genes, all the designed guides were much less efficient in reducing mRNA levels. However, even though complete silencing was not achieved, a very significant reduction in mRNA levels was found (>85%), and this can be sufficient for many experiments.

In addition, where genes that are crucial for cell survival are involved, cells do not survive with complete silencing, but this method might give cells with the highest level of transcriptional silencing they can tolerate. This allows for functional studies not possible using CRISPR-KO.

### Potential solutions

• In the first instance, using a higher MOI is recommended. There is a definite correlation between CRISPRi efficiency and MOI (see Figure 9).
• Design new guides that may achieve more efficient silencing.
• Investigate if there is a possibility of an alternative TSS. If so, one can design guides manually, using the CRISPR tracks (selecting green guides) around where the alternative site might be.

### Problem 5

*The guides are not regulating the same genes* (See design, cloning and production of CRISPRi lentiviruses)*.*

This would suggest that the guides are not specific and bind too many off-targets. In this work, the analysis of the guides was combined as described in step 4, in order to filter out genes that are regulated by only one of the guides and therefore not true targets (see also Johansson et al., 2022).

### Potential solutions

**Figure 10:**
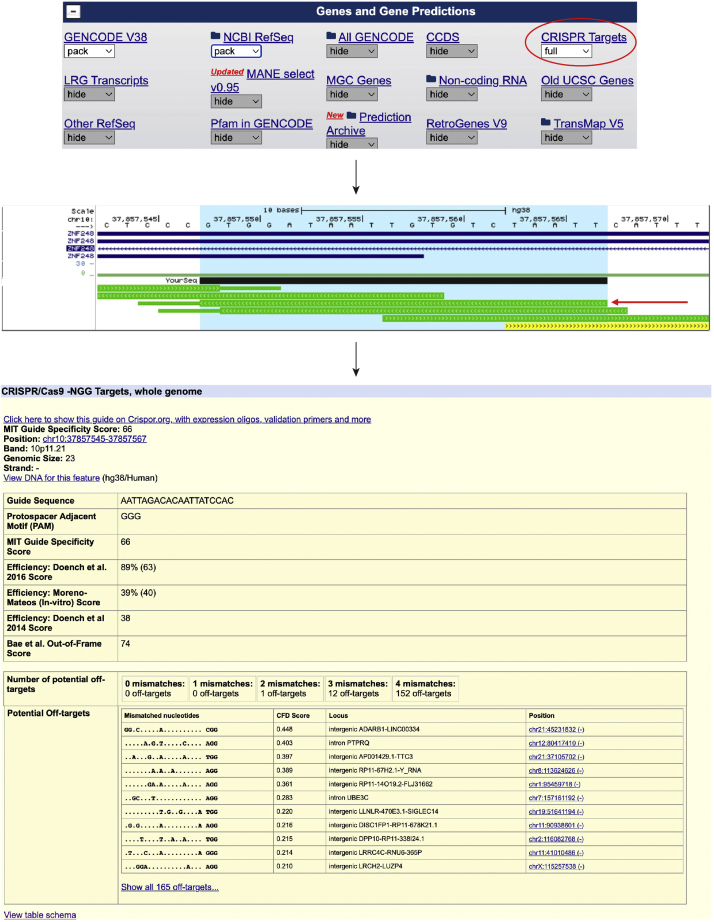
Investigation of off-targets for gRNAs

• Combine the analysis of both guides (control vs CRISPRi).
• Include a third guide in the analysis.
• Design new guides.
• Use in combination with another approach such as siRNA.
• Look for the expected off-targets for each guide (Figure 10).
  ◦ Go to UCSC Genome Browser.
  ◦ Selected correct genome.
  ◦ Enable CRISPR targets track.
  ◦ Paste in the guide sequence of choice.
  ◦ In the BLAST search window, confirm that the guide sequence only appears once in genome and press the browser link (see Figure 2).
  ◦ Highlight as described above and zoom out 1.5×.
  ◦ Click on the guide corresponding to the sequence (red arrow). The PAM sequence is seen as a thinner line in the browser track.
  ◦ All information about that guide, including predicted off-targets, can be found here.

Of the top 10 hits we recommend no overlap with potential off-targets. However, these are only potential off-targets and might not be affected and in addition other targets might be. We recommend handling off-targets with the use of combinatorial analysis, meaning only look at phenotypes seen with two guides.

## Resource availability

### Lead contact

Further information and requests for resources and reagents should be directed to and will be fulfilled by the lead contact, Dr. Pia Johansson (pia.johansson@med.lu.se).

### Materials availability

Plasmids generated in this study are available upon request.

## Data Availability

This study did not generate/analyze datasets or code.
